# P-687. Respiratory Syncytial Virus (RSV) Testing Patterns among Medically Attended Cases of Acute Respiratory Illness from 2016 to 2023

**DOI:** 10.1093/ofid/ofae631.883

**Published:** 2025-01-29

**Authors:** David Singer, Yan Wang, Aozhou Wu, Emily K Horn, Elizabeth M La, Susan Gerber, Joanna Boland, Keith A Betts

**Affiliations:** GSK, Philadelphia, Pennsylvania; Analysis Group, Los Angeles, California; Analysis Group, Los Angeles, California; GSK, Philadelphia, Pennsylvania; GSK, Philadelphia, Pennsylvania; GSK, Philadelphia, Pennsylvania; Analysis Group, Los Angeles, California; Analysis Group, Los Angeles, California

## Abstract

**Background:**

Historically, respiratory syncytial virus (RSV) has been an underrecognized cause of acute respiratory illness (ARI) in adults and patients have been infrequently tested for RSV. This study aimed to describe RSV testing patterns among adults over time and by setting of care.

**Figure:**
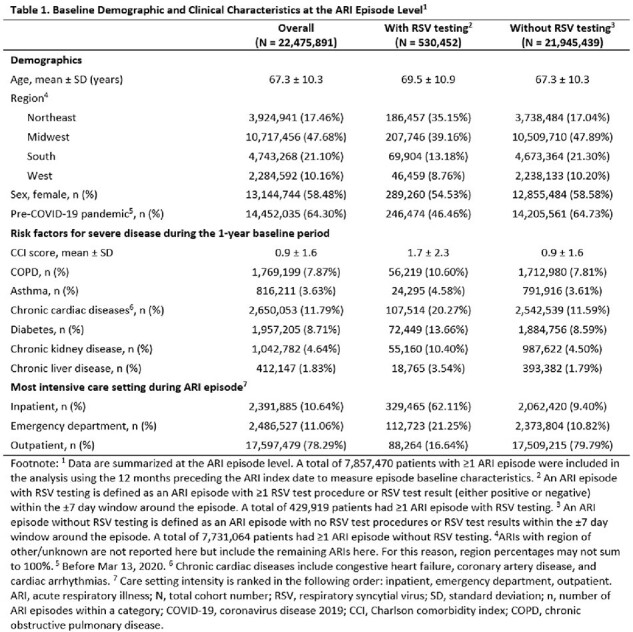

**Methods:**

This was a retrospective cohort study, analyzing the Optum de-identified Electronic Health Record data set (Optum EHR) from October 2015 to June 2023. Medically attended ARIs were identified (using ICD-10-CM codes) in adults aged ≥ 50 years with ≥ 12 months of activity in the Optum EHR. Individuals could have multiple ARIs included over the study period. ARIs were classified by epidemiological year (July 1 to June 30), RSV season, and by most intensive setting of care during the ARI (outpatient, emergency department [ED], or inpatient). The percentage of ARIs tested for RSV was reported by time period and other characteristics. Influenza and SARS-CoV-2 testing patterns were also reported for context.

**Figure:**
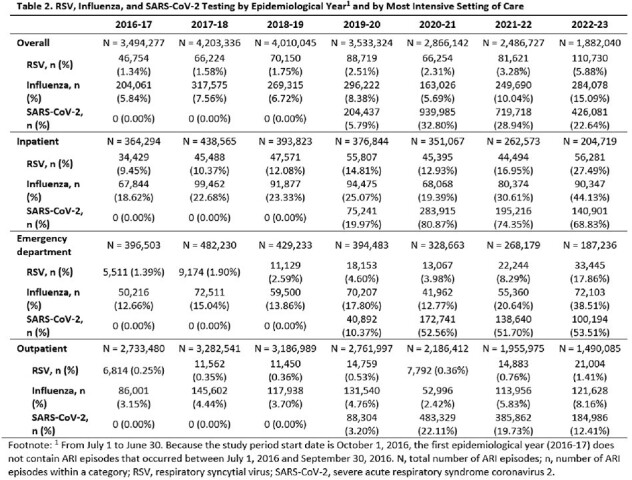

**Results:**

The study included 22,475,891 ARIs among adults aged ≥50 years. The mean age at the time of ARI was 67.3 years. Key baseline characteristics of ARI episodes are reported in Table 1. The percentage of ARIs tested for RSV generally increased each epidemiological year, from 1.3% in 2016-17 to 5.9% in 2022-23 (Table 2). RSV testing was higher during RSV seasons than outside of seasons prior to the COVID-19 pandemic period. Across epidemiological years, the percentages of medically attended ARIs tested for RSV was highest among ARIs that involved inpatient care (9.5-27.5%), followed by ED visits (1.4-17.9%), and outpatient visits (0.3-1.4%).

**Conclusion:**

Despite increased awareness of the importance of RSV as a pathogen in older adults and adults with comorbidities, RSV testing remains infrequent. RSV testing has increased over time, particularly among ARIs involving inpatient care. These findings suggest that studies relying on recent real-world data could substantially underestimate RSV burden. Future research may further explore ARI or patient characteristics associated with RSV testing.

FUNDING: GSK (study identifier: VEO-000617)

**Disclosures:**

**David Singer, PharmD, MS**, GSK: employee|GSK: Stocks/Bonds (Public Company) **Yan Wang, ScD**, Analysis Group: Employee **Aozhou Wu, PhD**, Analysis Group: Employee **Emily K. Horn, MSc**, GSK: employee|GSK: Stocks/Bonds (Private Company) **Elizabeth M. La, PhD**, GSK: employee|GSK: Stocks/Bonds (Private Company) **Susan Gerber, MD**, GSK: Employee **Joanna Boland, PhD, MS**, Analysis Group: Employee **Keith A. Betts, PhD**, Analysis Group: Employee

